# Ontological modeling and analysis of experimentally or clinically verified drugs against coronavirus infection

**DOI:** 10.1038/s41597-021-00799-w

**Published:** 2021-01-13

**Authors:** Yingtong Liu, Junguk Hur, Wallace K. B. Chan, Zhigang Wang, Jiangan Xie, Duxin Sun, Samuel Handelman, Jonathan Sexton, Hong Yu, Yongqun He

**Affiliations:** 1 Department of Computational Medicine and Bioinformatics, University of Michigan Medical School, Ann Arbor, MI 48109 USA; 2 University of North Dakota School of Medicine and Health Sciences, Grand Forks, ND 58202 USA; 3 Department of Pharmacology, University of Michigan Medical School, Ann Arbor, MI 48109 USA; 4 Department of Biomedical Engineering, Institute of Basic Medical Sciences and School of Basic Medicine, Peking Union Medical College and Chinese Academy of Medical Sciences, Beijing, 100005 China; 5 School of Bioinformatics, Chongqing University of Posts and Telecommunications, Chongqing, 400065 China; 6 Department of Pharmaceutical Sciences, College of Pharmacy, University of Michigan, Ann Arbor, MI 48109 USA; 7 Department of Internal Medicine, University of Michigan Medical School, Ann Arbor, MI 48109 USA; 8 U-M Center for Drug Repurposing, University of Michigan, Ann Arbor, MI 48109 USA; 9 Department of Medicinal Chemistry, College of Pharmacy, University of Michigan, Ann Arbor, MI 48109 USA; 10 Department of Respiratory and Critical Care Medicine, Guizhou Province People’s Hospital and NHC Key Laboratory of Immunological Diseases, People’s Hospital of Guizhou University, Guiyang, Guizhou 550002 China; 11 Department of Basic Medicine, Guizhou University Medical College, Guiyang, Guizhou 550025 China; 12 Unit for Laboratory Animal Medicine, University of Michigan Medical School, Ann Arbor, MI 48109 USA; 13 Department of Microbiology and Immunology, University of Michigan Medical School, Ann Arbor, MI 48109 USA

**Keywords:** Data integration, Classification and taxonomy, SARS-CoV-2, Viral infection

## Abstract

Our systematic literature collection and annotation identified 106 chemical drugs and 31 antibodies effective against the infection of at least one human coronavirus (including SARS-CoV, SAR-CoV-2, and MERS-CoV) *in vitro* or *in vivo* in an experimental or clinical setting. A total of 163 drug protein targets were identified, and 125 biological processes involving the drug targets were significantly enriched based on a Gene Ontology (GO) enrichment analysis. The Coronavirus Infectious Disease Ontology (CIDO) was used as an ontological platform to represent the anti-coronaviral drugs, chemical compounds, drug targets, biological processes, viruses, and the relations among these entities. In addition to new term generation, CIDO also adopted various terms from existing ontologies and developed new relations and axioms to semantically represent our annotated knowledge. The CIDO knowledgebase was systematically analyzed for scientific insights. To support rational drug design, a “Host-coronavirus interaction (HCI) checkpoint cocktail” strategy was proposed to interrupt the important checkpoints in the dynamic HCI network, and ontologies would greatly support the design process with interoperable knowledge representation and reasoning.

## Introduction

The COVID-19 outbreak, caused by SARS-CoV-2, has become a pandemic and is now spreading worldwide. As of September 26, 2020, over 32,586,000 confirmed cases with over 989,000 deaths, have been reported to WHO. In addition to COVID-19, two related betacoronavirus-induced diseases, including Severe Acute Respiratory Syndrome (SARS)^1^ and Middle East Respiratory Syndrome (MERS)^2^, had triggered public health crises. SARS emerged in China in November 2002, in an epidemic that lasted for 8 months and resulted in 8,098 confirmed human cases in 29 countries with 774 deaths (case-fatality rate: 9.6%)^1,3^. Approximately 10 years later in June 2012, the MERS-CoV, another highly pathogenic coronavirus, was isolated in Saudi Arabia from the sputum of a male patient who died from acute pneumonia and renal failure^2^. MERS-CoV outbreaks resulted in 2,260 cases in 27 countries and 803 deaths (35.5%)^4,5^. To successfully fight against future coronavirus infections, intensive studies are required to identify effective and safe measures against the current and past outbreaks.

Multiple studies^6–9^ aimed to discover and develop drugs targeted to these betacoronaviruses. These studies have achieved varying levels of success *in vitro* and *in vivo*. By integrating the results of these previous studies, we may find clues for the development and improvement of drugs to treat COVID19. Existing drugs might be repurposed for treating COVID-19, or patterns extracted from existing anti-coronavirus studies may lead to the development of new drugs.

In the informatics field, a formal ontology is a human- and computer-interpretable set of terms and relations that represent entities in a specific biomedical domain and their relationships. Ontology has played a significant role in knowledge and data standardization, integration, and analysis^10–13^. Coronavirus Infectious Disease Ontology (CIDO) is a community-based open source biomedical ontology that systematically represents entities associated with coronavirus diseases, including their etiological causes, phenotypes, host-coronavirus interactions, diagnosis, drugs, and vaccines^14^. For drug representation, CIDO reuses terms from three existing ontologies, including Chemical Entities of Biological Interest ontology (ChEBI)^15^, National Drug File – Reference Terminology (NDF-RT)^16^, and Drug Ontology (DrON)^17^, which have been frequently used for drug studies^18,19^. ChEBI is a database and ontology of over 56,000 molecular entities of biological significance with a focus on small chemical compounds. ChEBI ontologically classifies these compounds based on different categories such as structural and functional features. Produced by the U.S. Department of Veterans Affairs, NDF-RT organizes drugs in a hierarchical and formal representation by modeling drug characteristics including ingredients, chemical structure, physiologic effect, mechanism of action, and pharmacokinetics. DrON provides an ontological representation of the drug contents on the RxNorm terminology^20^ that contains all medications available on the US market. Once a list of drugs is identified, tools such as Ontofox^21^ can be used to extract these drugs and their related characteristics from an ontology and perform specific analyses^22–25^. The extracted terms can also be imported into CIDO, and new axioms can also be generated to interlink different terms as a way for computer-interpretable knowledge representation.

In this study, we report our systematic collection, annotation, and analysis of anti-coronavirus drugs from the biomedical literature. Over 130 chemical drugs and antibodies against human coronavirus diseases were identified. We mapped the majority of these drugs to the ontologies ChEBI^15^, NDF-RT^16^, and DrON^17^, and imported these terms to CIDO for further modeling and analyses. We applied ontology to categorize these drugs and used ontology-based bioinformatics methods to further analyze various features of these drugs. The gene/protein targets of these drugs were also retrieved, and drug-target networks were analyzed to identify hub drugs and drug targets. In the end, we will discuss how our results can be used to facilitate rational drug design for COVID-19.

## Results

### 151 anti-coronavirus drug compounds effective against viral entry, replication, and/or in stimulating host immunity

We manually collected and identified 151 chemicals drug compounds, each of which was tested in various cell lines *in vitro*, or *in vivo* in either patients or animal models. These drugs were all found effective against the infection of at least one human coronavirus, most of which were SARS-CoV, SAR-CoV-2, or MERS-CoV. These 151 anti-coronavirus drugs include: (i) 106 active drug compounds that can be mapped to at least one of the three ontologies: ChEBI, DrON, and NDF-RT (Supplemental Table 1), (ii) 14 drugs that do not have any record in these ontologies, and (iii) 31 biological drugs (which are all monoclonal or polyclonal antibodies) specifically targeting on coronavirus proteins (e.g., S protein) (Supplemental Table 2). These 106 drugs include many common drugs/chemicals for the treatment of SARS and MERS (Table 1). However, their protein targets are more diverse, which suggests potential diverse pathways to inhibit viral proliferation.

**Table 1:** Summary of experimentally or clinically verified results.

	# of antibodies	# of chemical drugs (DrON)	# of active chemical ingredients (ChEBI)	# of drug protein targets
SARS-CoV	7	49	68	93
MERS-CoV	19	43	62	83
SARS-CoV-2	5	10	14	17
Total*	31	67	99	165

*, the total numbers are not exactly the sums of the numbers in the columns since the total numbers do not count duplicates.

**Fig. 1:**
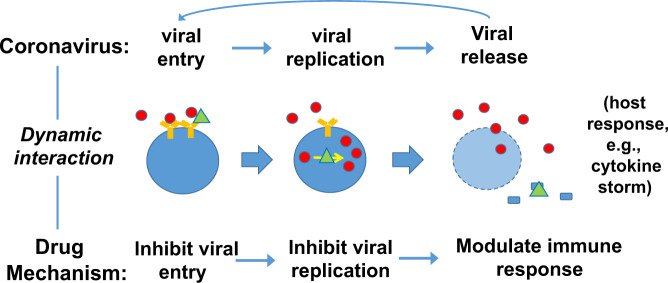
Modeling of the host-coronavirus interactions (HCIs) and HCI-drug interactions. The viruses enter into, survive in, and replicate in host cells. Correspondingly, drugs are developed to inhibit viral entry, replication, or modulate host immune responses. Red circles, yellow Y-shaped signs, green triangles, and blue blocks represent viruses, host receptors, drugs, and host immune factors (e.g., cytokines), respectively.

To better model and analyze the results collected, we first built a knowledge representation model (Fig. 1). Coronavirus infection lifecycle requires three processes: viral entry (or viral invasion), viral replication, and viral release. To fight against coronaviruses, drugs can function to interrupt any one of these steps. For example, an anti-coronaviral drug can interrupt the binding between the SARS-CoV-2 S protein and human ACE2 receptor and thus block the entry of the SARS-CoV-2 to human cells. Coronaviral infection can also cause many damages to the human cells and induce a series of host responses. Some anti-coronaviral drugs can modulate the host immune responses such as cytokine storm, thus preventing severe outcomes induced by these overreactive responses (Fig. 1).

Our study found that 32 drugs inhibiting viral entry to host cells, 51 drugs that inhibit viral replications inside host cells, and 11 drugs modulating host immune responses to coronavirus infection (Supplemental Table 1).

### CIDO representation of individual anti-coronaviral drugs, drug compounds, and their associated properties

CIDO was used as an ontological platform to systematically represent the information of drugs, chemicals, viruses, diseases, and other related entities. The knowledge was extracted from existing ontologies or annotated by our own literature mining and manual curation. Here, we represent how CIDO is being used to represent individual drugs, drug compounds, and other related properties. Remdesivir is used as a model drug for our presentation here.

As a drug used to successfully treat the first COVID-19 patient in the USA^26^, remdesivir has become a highly promising drug for treating COVID-19. Remdesivir is a nucleoside analog, which inhibits viral proliferation by inhibiting RNA-dependent RNA polymerase (RdRP, or Nsp12) (Fig. 1)^6^. It has been shown to effectively fight against several kinds of viruses, including SARS-CoV and MERS-CoV *in vitro*^7^. Its anti-viral effects were also identified in the rhesus macaque model infected with MERS-CoV^8^. A recent study showed remdesivir can inhibit SARS-CoV-2 infection *in vitro*^6^. Moreover, remdesivir has been evaluated in two clinical trials^27,28^, showing that the hospitalized COVID-19 patients receiving remdesivir treatment recovered faster than similar patients who received a placebo. As a result, the US Food and Drug Administration (FDA) has allowed remdesivir to be distributed and used to treat adults and children hospitalized with severe COVID-19 (https://www.fda.gov/media/137564/download). However, the utility of remdesivir for treating COVID-19 may be limited only to hospitalized patients due to rapid first-pass hepatic clearance when administered orally and thus requires infusion for adequate delivery^29^. Its usage may also require initiation before the peak of viral replication, which might not be feasible in the clinical situation^30^.

**Fig. 2:**
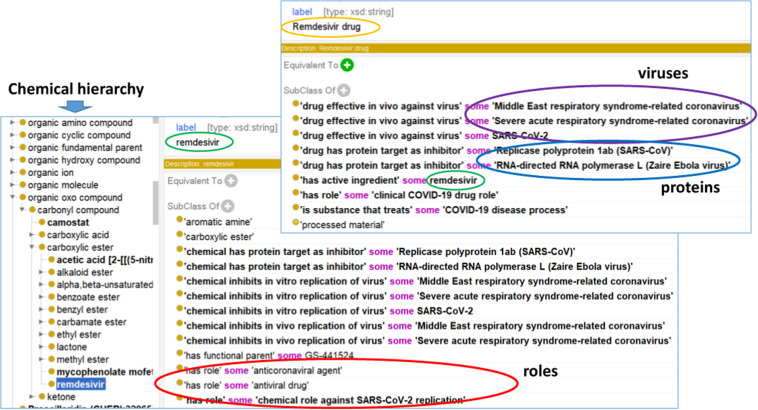
CIDO ontological representation of Remdesivir drug, remdesivir chemical, and their properties. The upper right screenshot is for the Remdesivir drug, which has the active ingredient of the remdesivir. The bottom screenshot is for the remdesivir, which is under a chemical hierarchy defined by ChEBI. Axioms are provided to define the relations between different entities including viruses, proteins, and roles.

Figure 2 demonstrates how CIDO represents remdesivir as a drug and remdesivir as a chemical entity. We used ChEBI as the default ontology for chemical entity representation. Initially, remdesivir was not in ChEBI. Therefore, we submitted a request and provided needed remdesivir information to the ChEBI development team. Eventually, this term was added to ChEBI. We then imported this term and other anti-coronaviral chemical entity terms as described earlier to CIDO. As defined in ChEBI, remdesivir is a carboxylic ester and has antiviral and anti-coronaviral agent roles. Furthermore, we added a new role to this drug, termed ‘chemical role against SARS-CoV-2 replication’ (Fig. 2). To link the chemicals with chemical targets or viruses, we have also generated new relations, such as ‘chemical inhibits *in vivo* replication of virus’, and used it to represent the relation between remdesivir and SARS-CoV-2:

*‘chemical inhibits in vivo replication of virus’ some SARS-CoV-2*

The above axiom shows that remdesivir can function to inhibit the replication of SARS-CoV-2 *in vivo*. As another example, CIDO uses the relation ‘chemical has protein target as inhibitor’ to represent how remdesivir is related to its protein target:

*‘chemical has protein target as inhibitor’ some ‘Replicase polyprotein 1ab (SARS-CoV)’*

This axiom shows that remdesivir can function as an inhibitor to inhibit the role of SARS-CoV replicase polyprotein 1ab^31^.

To represent remdesivir as a drug, we first imported this term from DrON to CIDO and added more annotations in CIDO (Fig. 2). Since this term from DrON is also labeled ‘remdesivir’, to avoid name duplication, we changed the name to ‘remdesivir drug’. A new axiom was added in CIDO to link the drug to its chemical ingredient:

*‘has active ingredient’ some remdesivir*

In addition, CIDO has generated new relations such as ‘drug effective *in vivo* against virus’ and ‘drug has protein target as inhibitor’ to link the drug to its viral targets or protein targets, respectively (Fig. 2).

### CIDO representation of host-coronavirus interactions (HCIs) and their interactions with individual drugs

**Fig. 3:**
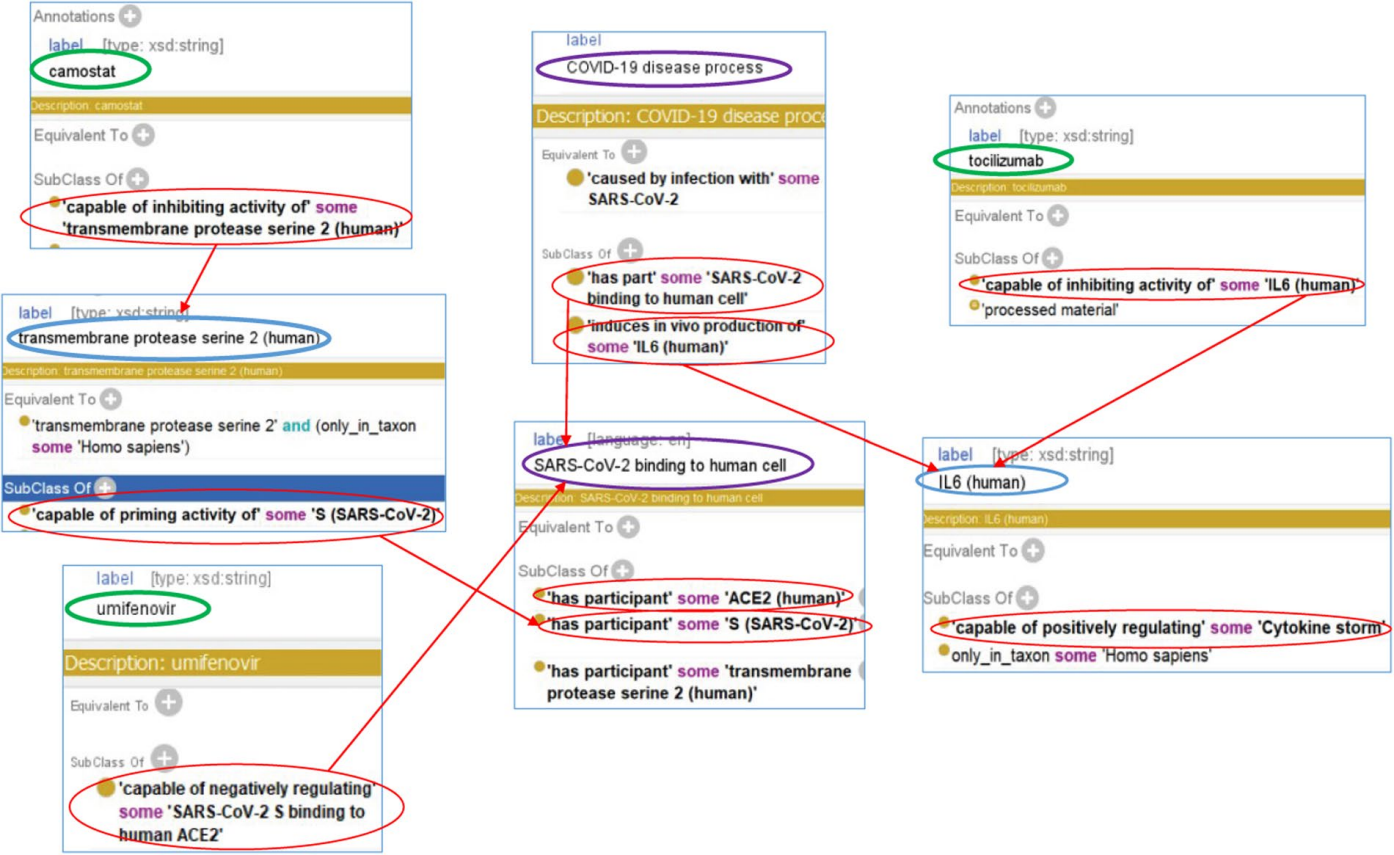
Ontological representation of HCIs and drugs targeting the interactions. Camostat and umifenovir are capable of inhibiting the viral entry to host cells. Tocilizumab is capable of inhibiting IL-6, a driver interleukin that mediates cytokine storm.

Figure 3 shows how CIDO represents three drugs including camostat, umifenovir, and tocilizumab and how these drugs participate in the processes against SARS-CoV-2 infection, the cause of COVID-19. SARS-CoV-2 enters the host cells through the binding between the viral envelope spike (S) glycoprotein and the host angiotensin-converting enzyme 2 (ACE2), and the S protein is primed by Transmembrane Serine Protease 2 (TMPRSS2)^32^. Such relation between TMPRSS2 and the S protein is represented as:

*TMPRSS2: ‘capable of priming activity of’ some ‘S (SARS-CoV-2)’*

Camostat, an inhibitor of TMPRSS2, can block the infection of SARS-CoV-2 in human lung cells^32^. Such action of camostat is then presented in CIDO as:

*‘capable of inhibiting activity of’ some ‘transmembrane protease serine 2 (human)’*

Using a similar strategy, CIDO models and presents the actions by umifenovir and tocizumab (Fig. 3). Umifenovir also works at viral entry level to inhibit coronavirus *in vitro*^33^ by interrupting the binding of ACE2 and the S protein.

To modulate host immune response, tocilizumab is capable of inhibiting the activity of IL-6, a critical mediator of cytokine storm (Fig. 3), which is an overreactive immune response that often occurs after SARS-CoV-2 infection^9^.

**Fig. 4:**
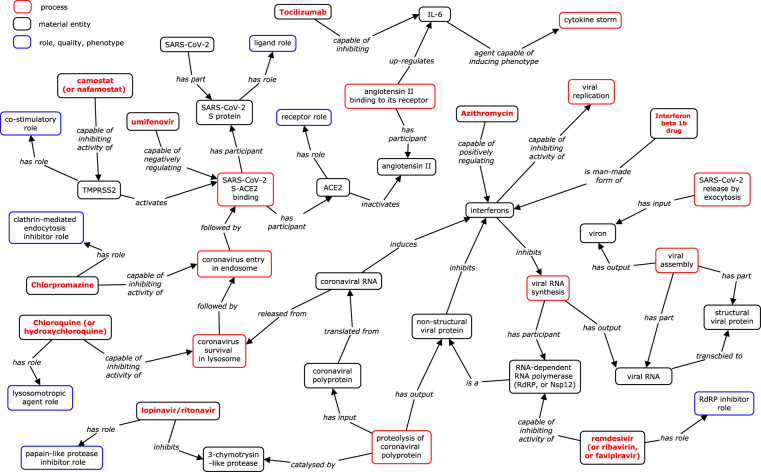
Ontological representation of host-coronavirus interactions and drugs targeting the interactions. The boxes enclosed in red, black, and blue colors represent biological processes, material entities (e.g., cells, molecules, and drugs), and roles or phenotypes, respectively. Red text in bold represents drugs. The text labeled in the middle of lines represents relations. The knowledge was obtained by our manual annotation of peer-reviewed publications.

Instead of the CIDO representation using the Protégé-OWL editor (Fig. 3), Fig. 4 provides a more classical representation of the HCIs and their interactions with individual drugs such as the three drugs presented above. Such representation is more understandable by researchers with no ontology background. We have also ensured that all the terms and linkages presented in the figure are also represented in CIDO.

Figure 4 also shows the mechanisms of many more drugs, including Lopinavir-Ritonavir, Ribavirin, and Interferon beta-1b. A triple combination of these three drugs in a clinical trial for treating mild to moderate COVID-19 patients was found to be safe and more effective than the usage of lopinavir-ritonavir alone^34^. These three drugs target different aspects of the whole life cycle of the disease, including the inhibition of the proteolysis of coronavirus polypeptides by Lopinavir-Ritonavir, inhibition of viral RNA synthesis by Ribavirin, and cytokine storm inhibition by Interferon beta-1b (Fig. 4).

### Drug-target network and ontological drug-target interactions analysis

**Fig. 5:**
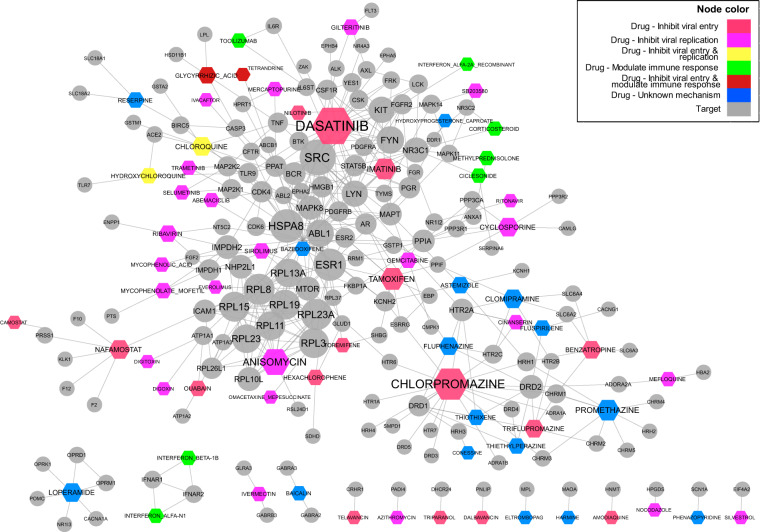
Drug-host target network. The network contains 48 unique drugs (hexagons) and 163 their known targets (circles collected primarily from DrugBank) with 428 interactions. Drugs without any known human protein targets were excluded. Node size corresponds to the number of connections each node has, also known as degree. Node color indicates different types of drugs or drug targets (gray).

A drug-target interaction network (Fig. 5) was generated to include all unique drugs and their known targeted proteins according to the records from DrugBank. This network included 68 drugs with 163 known human protein targets with a total of 428 interactions. Multiple clusters were identified from visual inspection of this network. The biggest one included the majority of the drugs inhibiting viral replication (nodes in violet) and modulating immune response (nodes in green). Another one was centered on the drug chlorpromazine as well as many drugs with unknown mechanisms with respect to their usages in coronavirus treatment.

Three drugs with the most connections in Fig. 5 are chlorpromazine, dasatinib, and anisomycin. Chlorpromazine has a function or mechanism of action (MoA) as a dopamine antagonist and adrenergic-alpha antagonist in NDF-RT. Our experimental study also found that metoclopramide and domperidone, both dopamine d2 receptor antagonists, had excellent efficacy against SARS-CoV-2 infection^35^. Chlorpromazine also interacts with the serotonin receptors (HTRs) and histamine receptors (HRHs) (Fig. 5). Based on ontology analysis, chlorpromazine functions as an antagonist or inhibitor of multiple dopamine receptors, adrenergic receptors, and histamine receptors. Although how chlorpromazine exhibits anti-coronavirus properties is not fully understood, adrenergic antagonists and histamine antagonists have been shown to inhibit RNA viruses such as Ebola and Marburg viruses^36^. As another hub drug, dasatinib has been annotated for over 20 protein targets and approximately half of them belong to tyrosine kinase proteins. Dasatinib, together as imatinib (another coronavirus drug), is an inhibitor of the Abelson murine leukemia viral oncogene homolog 1 (ABL1) pathway, a signaling pathway involved in cell differentiation, cell adhesion, and cellular stress response. Previous studies showed that dasatinib and imatinib can both inhibit BCR-ABL interaction and prohibit virus fusing with S protein of host cells^37,38^. As the 3^rd^ hub, anisomycin has 13 annotated protein targets in our ontological representation. Although there are no annotations for the role it plays in these interactions, it may be involved in protein translation since 10 of the protein targets are ribosomal proteins (Fig. 5).

### Key drug-targeted biological pathways identified from GO term enrichment analysis

**Fig. 6:**
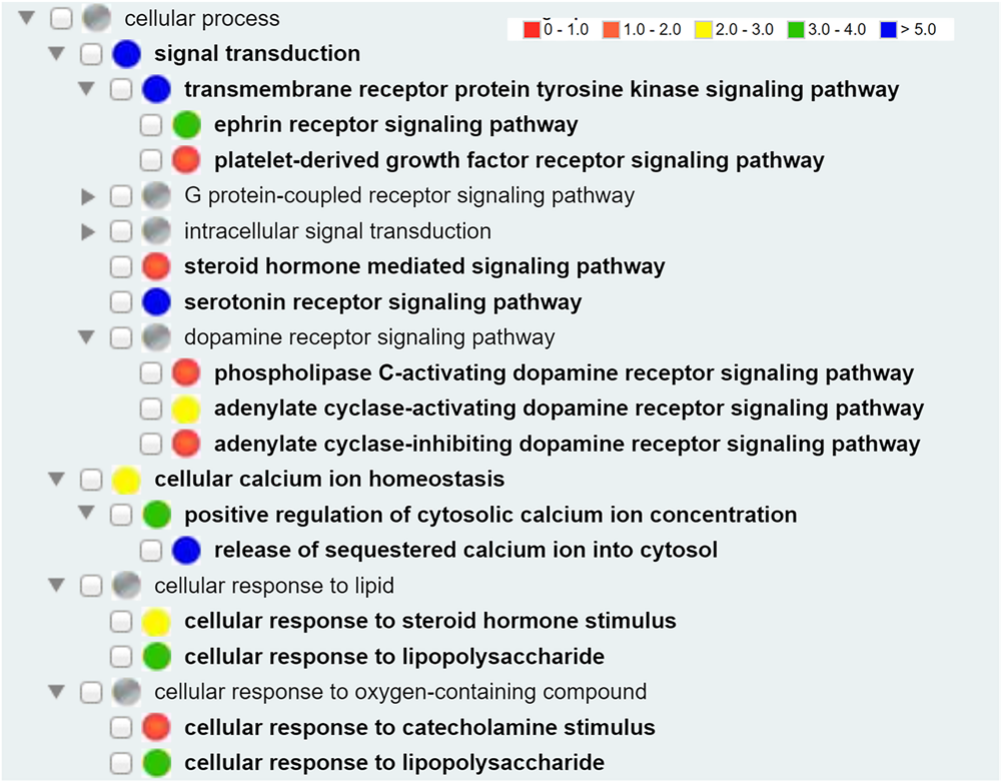
Hierarchical display of enriched GO biological process terms in drug targets. A total of 126 GO biological process terms were found significant with a false discovery rate (FDR) cut-off of 0.05 and different colors of the circles indicate the p-values ranges. The GOfox tool was used for the visualization. Only a selective set of terms are displayed here.

Our GO-based analysis on 147 human proteins identified 125 GO biological process terms significantly enriched at false discovery rate (FDR) < 0.05. Many signaling pathways such as signaling pathways mediated by ephrin receptor, steroid hormone, and serotonin were enriched (Fig. 6). Dopamine receptor signaling pathway appears important to the COVID-19 disease process, as indicated by enriched phospholipase C-activating, adenylate cyclase-activating, and adenylate cyclase-inhibiting dopamine receptor signaling pathways. Both positive and negative regulations of cytosol calcium concentrations were also identified, suggesting the important role of cytosol calcium in the disease progression. Cellular responses lipids and oxygen-containing compounds were also identified in our study. Note that we also performed KEGG pathway enrichment analysis with similar results (Supplemental Fig. 1).

To support integrative representation and analysis, the 125 enriched GO biological process terms were also extracted from GO and imported to CIDO. Furthermore, tyrosine-protein kinase Lck (human), a protein target of the drug dasatinib, participates in the GO ‘release of sequestered calcium ion into cytosol’ (GO_0051209). CIDO logically represents the information using the following axioms:

*dasatinib: ‘drug has protein target’ some ‘tyrosine-protein kinase Lck (human)’*

*tyrosine-protein kinase Lck (human): ‘participates in’ some ‘release of sequestered calcium ion into cytosol’*

Using these axioms, CIDO allows the logical interconnection between drugs, drug targets, and biological processes that involve these drug targets. Such interlinks support more advanced data and knowledge query and analysis.

### CIDO-based semantic query of anti-coronavirus drug knowledge

**Fig. 7:**
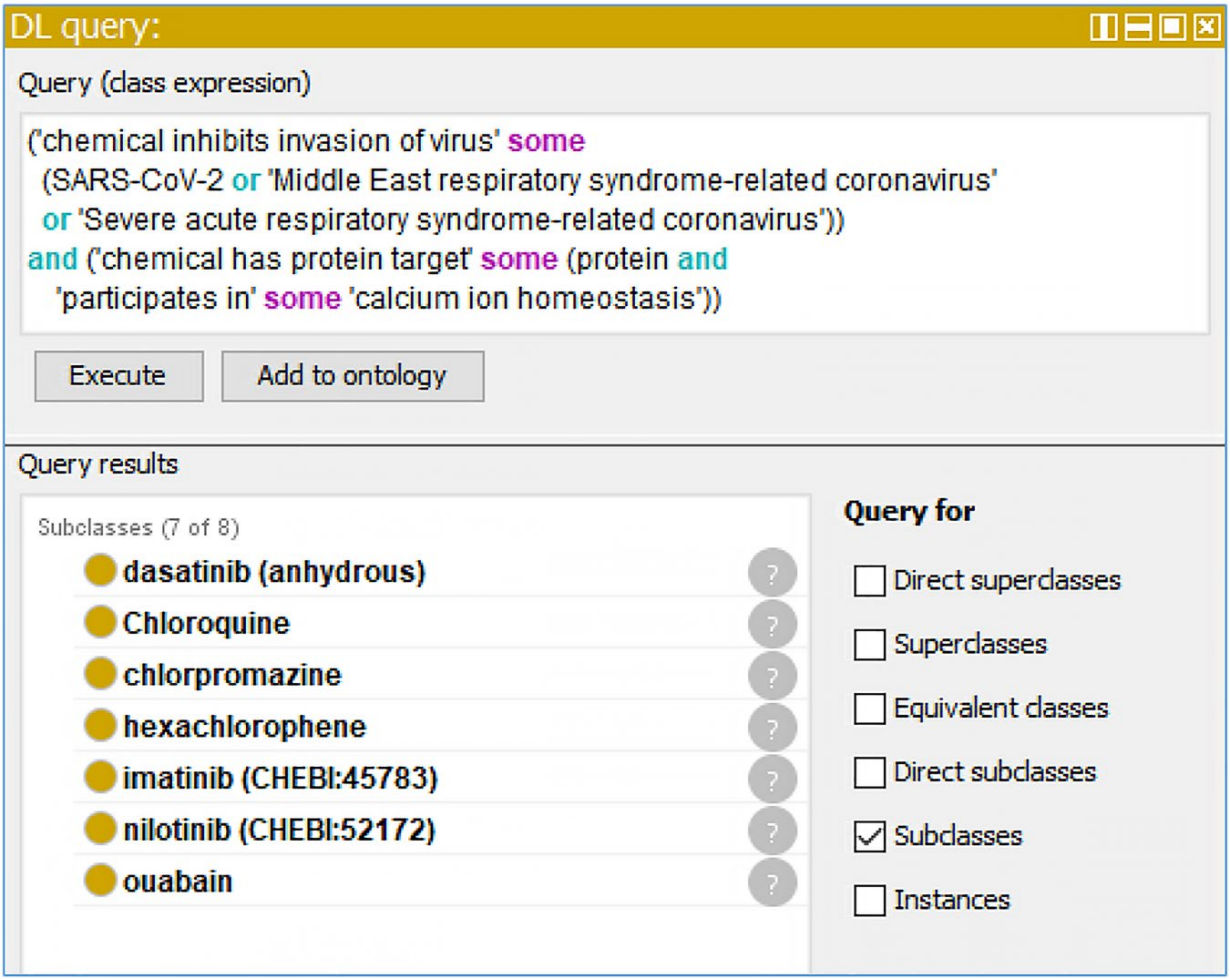
A DL query example. This query identified 7 chemicals that are capable of inhibiting the invasion of three human coronavirus, and are able to inhibit some known protein(s) which participate in calcium ion homeostasis biological process.

The CIDO ontology is formatted using the Web Ontology Language (OWL; https://www.w3.org/OWL/), a computer-interpretable Semantic Web language designed to represent rich and complex knowledge about things and relations between them. The CIDO ontology can be queried using different approaches such as Description Logic (DL) queries or SPARQL Protocol and RDF Query Language (SPARQL; https://www.w3.org/TR/rdf-sparql-query/). Figure 7 demonstrates how we can apply DL-query to search from CIDO the drugs that fit our defined criteria. We narrowed our query range to chemicals that prohibit any one of SARS-CoV-2, MERS, and SARS by inhibiting at viral entry level, and that also have protein target(s) participating in calcium ion homeostasis. This query identified seven drugs, including dasatinib, chloroquine, chlorpromazine, hexachlorophene, imatinib, nilotinib, and ouabain (Fig. 7). The DL query can also be updated depending on our needs, such as by constricting target viruses, target proteins, chemical properties, drug properties, and experimental type (*in vitro* or *in vivo*). SPARQL can also be used (Supplemental Fig. 2).

## Discussion

There are several aspects as to how the present study is novel. First, it presents the systematic collection and analysis of experimentally or clinically verified drugs against the infection of human coronaviruses *in vivo* or *in vitro*. Second, we demonstrated how ontologies could be leveraged to systematically analyze the drug information. Third, CIDO was used as an ontological platform to model and represent the complex and rich information about anti-coronaviral drugs, drug compounds, drug targets, biological processes, and the relations among these entities. Ontologies can logically, hierarchically, and systematically represent various drugs and their characteristics. Therefore, the usage of ontologies significantly enhanced our analysis.

With different chemical structures, targets and functions, our collected anti-coronavirus drugs target various aspects and stages of the viral life cycle and the interactions between coronaviruses and hosts (Fig. 1). The viruses strive to invade host cells, replicate there, and then release outside to infect new cells. Effective drugs usually target one or more stages of the viral cycle or alleviate overreactive host responses such as a cytokine storm.

While how drugs interact with hosts and viruses is complex, ontologies provide a scalable and interoperable platform for modeling, standardization, sharing, and analysis of the rich information. CIDO is an interoperable ontology that reuses many terms from existing ontologies including ChEBI, DrON, NDF-RT, PR, and GO, and seamlessly aligns and incorporates these many terms together into a comprehensive and integrative system. In addition to the ontology term import and reuse, CIDO also generated many new relations and axioms to build up computer-understandable knowledge for further reasoning and applications.

Given there are still no effective drugs for the robust treatment of COVID-19 patients, it is well recognized that drug combinations are needed to properly treat COVID-19. As an example of drug combinations, a triple combination of Interferon beta-1b, Lopinavir-Ritonavir, and Ribavirin in the treatment of mild to moderate COVID-19 patients was found to be safe and superior to lopinavir-ritonavir alone in alleviating symptoms and shortening the duration of viral shedding^34^. Several studies have shown that lopinavir (an enzyme inhibitor) can have favorable outcomes in treating SARS and MERS in combination with ritonavir (another enzyme inhibitor) in human and nonhuman primates^39,40^. A case study showed that the lopinavir/ritonavir combination significantly decreased viral load on one COVID-19 patient in Korea^41^. However, a clinical study with a total of 199 adult COVID-19 patients showed no significant benefit in improving clinical outcomes^42^. Human coronaviruses can delay IFN induction and dysregulate Interferon-stimulated gene (ISG) effector functions in primary human lung epithelial cells^43,44^. IFN beta 1b was also found to decrease virus-induced lung fibrosis in mice^45^. The usage of IFN beta 1b appeared to have a synergistic role in combination with Lopinavir-Ritonavir and Ribavirin to treat COVID-19 patients^34^. Such a cocktail therapy has been successful in treating HIV patients^46,47^. The combination drug treatment known as the “AIDS cocktail” or highly active antiretroviral therapy (HAART) was initiated in 1995, and since then has made AIDS a manageable disease.

Here we would like to propose an “HCI checkpoint cocktail” strategy that targets to interrupt the important checkpoints in the dynamic host-coronavirus interaction (HCI) network. Inspired by the immune checkpoint theory for cancer immunotherapy^48^, the checkpoint here denotes a key point in the dynamic HCI network where a major biological and/or regulatory process is performed. For COVID-19, the host-coronavirus interaction network includes two parts: coronaviral pathogenesis and host immune responses; each part is indeed a network containing many checkpoints. Coronavirus pathogenesis results in the uncontrollable viral entry, replication, and release (Fig. 1). Host responses help many infected patients but fail to help susceptible patients. Overreactive host responses such as cytokine storm even harm patient health. Our checkpoint cocktail strategy requires our careful investigation of the checkpoints and identifies ways to interrupt or block the major checkpoints under different conditions.

Specific checkpoints of coronaviral pathogenesis can be found in the viral life cycle. The major viral checkpoints include: viral binding and entry to the host cell, release from lysosomes, RNA expressed to protein, viral assembly, and viral release from host cells. Many drugs can be used to block the major checkpoints in the viral life cycle. For example, camostat and umifenovir (sold under brand name Arbidol) can be used to block the S-ACE2 binding, chlorpromazine inhibits viral entry from endosome, chloroquine, and hydroxychloroquine inhibit viral survival in lysosome, lopinavir inhibits the proteolysis of viral polypeptides and formation of non-structural proteins, and remdesivir inhibits RdRP and viral RNA synthesis (Fig. 4).

We further propose an ontology-based HCI checkpoint cocktail development strategy, in which ontology is significantly important in advancing the rational design of the “HPI checkpoint cocktail” strategy. Ontology has its own unique advantages in systematically and logically representing the checkpoints and how they interact with other entities such as cells, drugs, biological processes, and diseases. Moreover, it can be greatly used to aid in the modeling, representation, integration, and analysis of the dynamic HCI network, HCI-drug interaction, and rational development of repurposed drugs against viral infections. Utilization of an ontology serves a unique role in providing standard and computer-understandable representation on entities in the host-virus interactions and logic relations among these entities. We plan to use CIDO to further analyze various entities and relations in the drug-coronavirus-host interaction network, intending to make it an ontology backbone of knowledge representation and reasoning. We also look forward to developing new ontology-based algorithms and bioinformatics tools that further support artificial intelligence-based data analysis, testable hypothesis generation, and rational design of drug cocktails for effective and safe treatment of COVID-19 and possible future coronavirus diseases.

To effectively apply the “HPI checkpoint cocktail” strategy for COVID-19 drug cocktail design, it would be good to develop a user-friendly dedicated tool so that non-experts can easily come up with their own cocktail hypotheses. We are currently in the process of developing such a tool. Before such a tool is available, the researchers can use our manually annotated results in Excel or CSV format, which has categorized the drug mechanisms in different groups. Meanwhile, the COVID-19 literature is growing and the results are being updated at an unprecedented rate. We commit to periodical updates to provide an up-to-date version of the experimentally confirmed and well-annotated results.

While SARS-CoV, SARS-CoV2, and MERS-CoV are all coronaviruses, their responses to drugs in humans may overlap but still differ significantly. For example, while they all use S protein as the ligand, SARS-CoV and SARS-CoV-2 S proteins bind to the ACE-2^49,50^, but MERS-CoV S protein binds to human dipeptidyl peptidase 4 (DPP4; CD26) receptors^51^. Therefore, ACE-2 receptor blockers become a therapeutic approach for COVID-19^52^, and anti-DPP4 (CD26) is a therapeutic option for fighting MERS-CoV^53,54^. SARS-CoV and SARS-CoV-2 also have different immune responses in humans. For example, compared with SAR-CoV, SARS-CoV-2 did not significantly induce types I, II, or III interferons in *ex vivo* infected human lung tissues^55^. The biological processes stimulated by these three viruses in the hosts also differ in many ways^56^. Therefore, more research is required to further explore in depth the subtle differences of these viruses in their induced immune responses and drug effects.

The drug targeted proteins participate in different biological processes. To further identify those important biological processes having participants of the drug targeted proteins, we performed a GO enrichment analysis using the BioGRID interactome as the background. It is noted that the results of our GO enrichment analysis led to the discovery of correlations that might not be causal. A recent study performed comparative viral-human protein-protein interaction (PPI) and viral protein localization analysis for SARS-CoV-2, SARS-CoV, and MERS-CoV, and detected host factors that functionally inhibit coronavirus proliferation^56^. Their study identified 332 SARS-CoV-2-human PPIs and 67 druggable human proteins targeted by 69 existing FDA-approved drugs, drugs in clinical trials, and preclinical compounds^56,57^. The drugs collected by their study^56,57^ were based on protein-drug interactions, which is different from our drug collection based on drug-virus interaction. We will systematically annotate and incorporate their results into our ontology-based representation and analysis in the future.

## Methods

### Literature annotation and data extraction of anti-coronavirus drugs

Peer-reviewed articles in PubMed, Google Scholar, and PubMed Central literature databases were searched using relevant keywords, including coronavirus, SARS, MERS, COVID-19, drug, therapy, and medicine. Chemical or biological drugs that exhibited anti-coronavirus properties in lab settings were collected from research papers published until May 17, 2020. To be included in our list, each drug was required to demonstrate a significant level of viral inhibition *in vitro* or *in vivo*. For *in vitro* studies, all drugs exhibiting some extent of inhibition (EC_50_ > 0) are collected. Drugs in clinical studies showing statistical significance (≤0.05) are included. For each identified drug, we recorded its targeted virus, mechanism, experimental model, assay, and paper citation(s). Antibodies mentioned in this literature were also recorded with their types and antigens.

### Ontology extraction and analysis

The list of identified anti-coronavirus drugs was mapped to ontology IDs from ChEBI^15^, NDF-RT^16^, and DrON^17^. The Ontobee ontology repository^58^ was used for the mapping. Using the ontology IDs collected above as input, we extracted subsets of these three ontologies by the ontology extraction tool Ontofox^21^. The output ontologies are in the format of OWL. Protégé 5.0 OWL ontology editor (http://protege.stanford.edu/) was used for ontology editing and analysis. The annotated data are stored at the GitHub website: https://github.com/CIDO-ontology/anti-coronavirus-drugs. The GitHub website hosts the information of the community-based Coronavirus Infectious Disease Ontology (CIDO)^14^, which is targeted to include the annotated drug information out of this study. Since ontology is computer-understandable, we can query the ontology using different approaches including SPARQL and Description Logic (DL) query.

### Annotation of drug targets and drug-target network

The known targets of the identified drugs were collected from DrugBank^59^. For any drug without a matching DrugBank record, we relied on multiple other online resources, including ChEMBL and Wikipedia, to identify any known targets. Drug-target and protein-protein interactions among these targets, collected from the BioGRID interaction database^60^, were used to construct a drug-target interaction network and visualized using Cytoscape v3.7.2^61^. The collected drug targets were subjected to a pathway enrichment analysis using our in-house functional enrichment tool richR (http://hurlab.med.und.edu/richR) in terms of the Kyoto Encyclopedia of Genes and Genomes (KEGG) pathways^62^. The FDR adjusted p-values were calculated, and the cutoff the 0.05 was applied for statistical significance measurement.

### Gene ontology (GO) term enrichment analysis and representation

DAVID Bioinformatics Resources^63^ was used for GO term enrichment analysis to find significantly enriched biological processes by the drug-targeted proteins using the human interactome obtained from the BioGRID database as the background. BioGRID GOfox (http://gofox.hegroup.org/)^64^ was used to visualize the hierarchical results of the enriched GO terms. The FDR adjusted p-values were calculated with the cutoff the 0.05 used for statistical significance measurement.

### Further CIDO modeling and analysis of collected anti-coronavirus drugs

To systematically connect all ontologies and include more information, we imported the Ontofox-extracted terms from ChEBI, DrON, and NDF-RT to CIDO and aligned these terms with the overall CIDO design^14^. In addition, CIDO includes newly identified drug targets as defined in the Protein Ontology (PR)^65^, and the enriched biological processes as defined in the Gene Ontology (GO)^10^. New relations were also generated to interlink different CIDO components including diseases, drugs, chemicals, drug targets, and biological processes. Ontorat^66^ was used for the high-throughput generation of new relations in CIDO.

## Supplementary information

Supplemental Figures

Supplemental Tables

## Data Availability

The data and materials introduced are all openly available in this article or at the GitHub website: https://github.com/CIDO-ontology/anti-coronavirus-drugs. The data files are provided in Excel and CSV formats. The data explanation is also provided on the GitHub website. Meanwhile, these data have also been stored in the OSF data repository (10.17605/OSF.IO/7TD94)^67^.

## Code availability

As an OBO library ontology (http://www.obofoundry.org/ontology/cido.html), the code of CIDO is openly available: http://purl.obolibrary.org/obo/cido.owl. The license of the code is Creative Commons BY 4.0. The source code uses the license CC-BY. CIDO has been deposited to the Ontobee ontology repository (http://www.ontobee.org/ontology/CIDO), the BioPortal repository (https://bioportal.bioontology.org/ontologies/CIDO), and the OLS repository (https://www.ebi.ac.uk/ols/ontologies/cido).
