# Supplementary material for: Ontological modeling and analysis of experimentally or clinically verified drugs against coronavirus infection
Source: Sci Data. 2021 Jan 13;8:16. doi: 10.1038/s41597-021-00799-w (PMC7806933; doi:10.1038/s41597-021-00799-w)
Supplement: Supplementary file 1 — Supplemental Figures [file 41597_2021_799_MOESM1_ESM.docx]

**SUPPLEMENTAL FIGURES:**


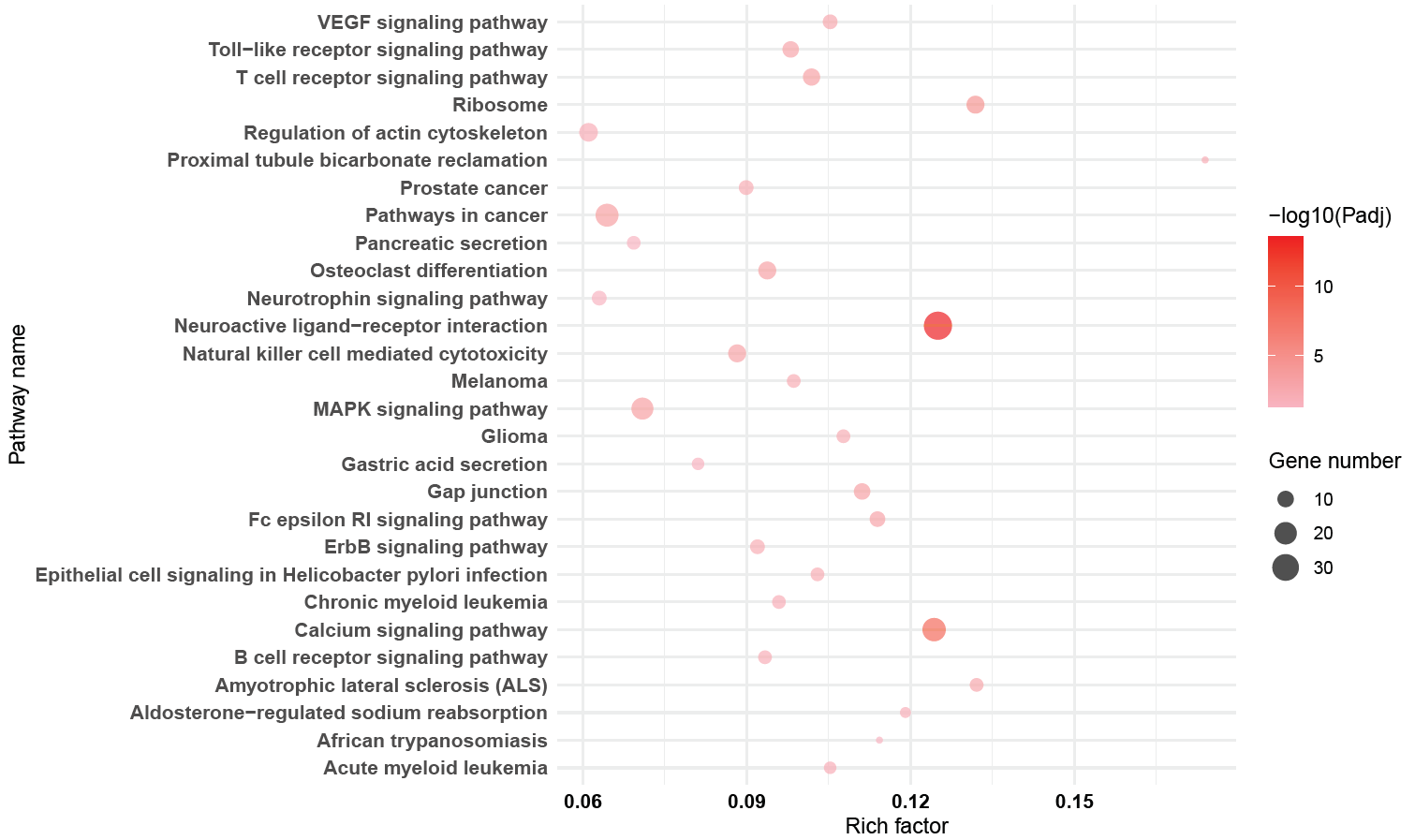


**Supplemental Fig. 1. Significantly enriched KEGG pathways among the known host targets of anti-coronavirus drug.** Our in-house enrichment analysis R package richR was used to perform the enrichment analysis and generate a dot plot. This plot includes the 28 significantly enriched KEGG pathways with adjusted p-value < 0.05. The rich factor is the proportion of known drug targets to all genes in the human genome belonging to each KEGG term. The size of the dot corresponds to the numbers of known drug targets annotated with the corresponding KEGG pathways. The color gradient (see color scale right of the figure) indicates the level of significance, represented by –log10(Padj), where the P-value was adjusted with false discovery rate (FDR).


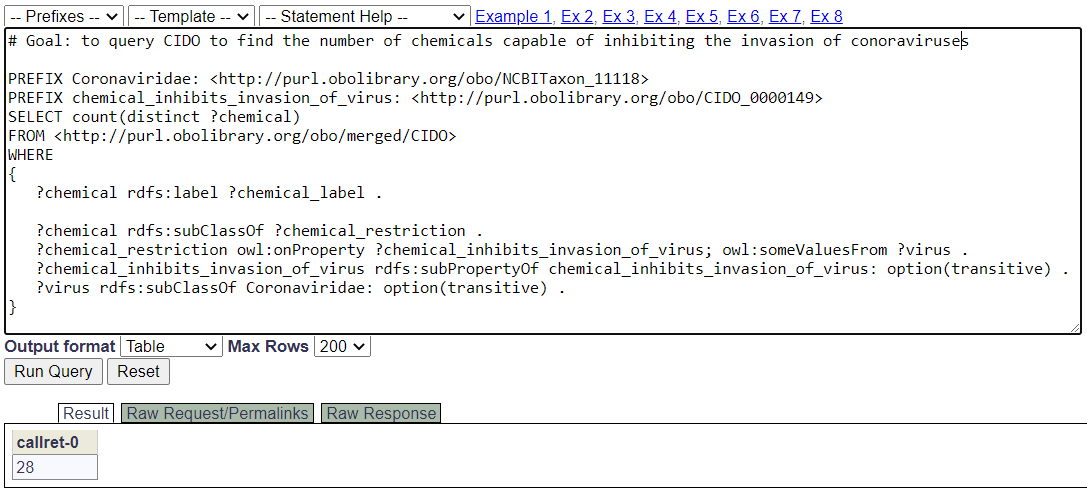


**Supplemental Fig. 2. SPARQL query demonstration.** This SPARQL query identified 28 chemicals that are capable of inhibiting the invasion of coronaviruses *in vitro* or *in vivo*. The query was performed using the Ontobee SPARQL endpoint (<http://www.ontobee.org/sparql>).
