# Supplementary material for: Ontological modeling and analysis of experimentally or clinically verified drugs against coronavirus infection
Source: Sci Data. 2021 Jan 13;8:16. doi: 10.1038/s41597-021-00799-w (PMC7806933; doi:10.1038/s41597-021-00799-w)
Supplement: Supplementary file 2 — Supplemental Tables [file 41597_2021_799_MOESM2_ESM.docx]

**SUPPLEMENTAL TABLES:**

**Supplemental Table 1.** Anti-coronavirus drugs with experimental data support

| **Chemical Ingredients**  **(Drug name)** | **Targeted virus(es)** | **Ontology IDs** | **PUBMED IDs** |
| --- | --- | --- | --- |
| ***Inhibit viral entry*** | | | |
| Aloxistatin (E-64d) | S2 | C: 101381 | 32142651 |
| Amodiaquine | S | C: 2674, D: 00012403, N: N0000147704,  DB00613 | 32366720, 30700611 |
| Arbidol | S2 | C: 134730, DB13609 | 32373347 |
| Benztropine mesylate | S, M | C: 3049, D: 00059818, N: N0000146182,  DB00245 | 26202243, 24841273 |
| Bufalin | M | C: 517248 | 26868298 |
| Camostat | S, M | C: 135632, DB13729 | 28855003, 25666761 |
| Camostat mesylate | S2 | C: 135632, DB13729 | 32142651 |
| Chloroquine | S, M, S2 | C: 3638, D: 00001135, N: N0000147767, DB00608 | 16837072, 27344959,  27916837, 32150618, 32020029 |
| Chloroquine phosphate | S, M, S2 | N: N0000146559, DB00608 | 24841273, 32150618, 32074550 |
| Chlorpromazine | M, S2 | C: 3647, D: 00021685, N: N0000146214, DB00477 | 27344959, 24841269, 32387014 |
| Chlorpromazine hydrochloride | S, M | C: 3649, D: 00061920, N: N0000146213,  DB00477 | 24841273 |
| Dalbavancin | S, M | C: 82721, N: N0000171775, D: 00750811, DB06219 | 26953343, 28855003, 16081529 |
| Dasatinib | S, M | C: 49375, D:00018901, N: N0000176043,  DB01254 | 24841273, 26868298 |
| Hexachlorophene | S | C: 5693, D: 00014863, N: N0000146582,  DB00756 | 32366720, 31027241 |
| Hydroxychloroquine | S2 | C: 5801, N: N0000147871, D: 00010111, DB01611 | 32150618, 32205204 |
| Hydroxychloroquine sulfate | S, M | D: 00061833, N: N0000146583, DB01611 | 32150618, 24841273 |
| Imatinib | S, M | C: 45783, D: 00018693, N: N0000148698, DB00619 | 30711575 |
| Imatinib mesylate | S, M | C: 31690, N: N0000148699, DB00619 | 24841273 |
| Nafamostat | S, M | C: 135466, DB12598 | 28855003,30711575,  27550352 |
| Nafamostat mesylate | S2 | C: 31890, DB12598 | 32020029 |
| Nelfinavir mesylate | S2 | C: 7497, N: N0000148479, DB00220 | 32374457 |
| Nilotinib | S | C: 52172, N: N0000176124, D:00018985, DB04868 | 24841273, 29557770 |
| Oritavancin | S, M | C: 82699, D: 00750820, DB04911 | 26953343, 28855003, 16081529 |
| Ouabain | S, M | C: 472805, D:00015446, DB01092 | 26868298 |
| Tamoxifen citrate | S, M | C: 9397, D: 00075906, N: N0000146786,  DB00675 | 23785035, 24841273 |
| Teicoplanin | S, M | D: 00012681, DB06149 | 26953343, 28855003 |
| Telavancin | S, M | C: 71229, N: N0000180310, D: 00019459, DB06402 | 26953343, 28855003, 16081529 |
| Terconazole | S, M | C: 9451, D: 00013976, N: N0000147638, DB00251 | 30893774, 24841273 |
| Toremifene citrate | S, M | C: 9636, N: N0000148546, DB00539 | 23785035, 24841273 |
| Triflupromazine hydrochloride | S, M | C: 9712, N: N0000146110, DB00508 | 24841273 |
| Triparanol | S, M | C: 135714, N: N0000166394 | 30893774, 24841273 |
| Valinomycin | S | C: 28545, N: N0000170352, DB14057 | 30858482, 16837072 |
| ***Inhibit viral replication*** | | | |
| 6-mercaptopurine | S, M | C: 50667, N: N0000006010, DB01033 | 27344959, 28855003 |
| 6-thioguanine | S, M | C: 9555, DB00352 | 27344959, 28855003 |
| Abemaciclib | S | D: 00803385, DB12001 | 32366720 |
| Anisomycin | S, M | C: 338412, N: N0000167149, DB07374 | 24841273 |
| Arbidol | S2 | C: 134730, DB13609 | 32373347 |
| Azithromycin | S2 | C: 2955, N: N0000148074, D: 00024808, DB00207 | 32205204 |
| Cepharanthine | S, O | C: 3546 | 32366720, 31690059 |
| Berbamine | S | C: 3063 | 32366720, 29305616 |
| Chloroquine | S, M, S2 | C: 3638, D: 00001135, N: N0000147767, DB00608 | 16837072, 27344959,  27916837, 32150618, 32020029 |
| Chloroquine phosphate | S, M, S2 | N: N0000146559, DB00608 | 24841273, 32150618 |
| Cinanserin | S | N: N0000166641 | 16837072 |
| Cycloheximide | S, M | C: 27641, N: N0000167211 | 24841273 |
| Cyclosporine | S, M | C: 4031, D: 00023979, N: N0000147064 DB00091 | 27478032, 27344959 |
| Digitoxin | S | C: 28544, D: 00016250, N: N0000145817, DB01396 | 32366720, 29321306 |
| Digoxin | S | C: 4551, D: 00012840, N: N0000146388,  DB00390 | 32366720 |
| Everolimus | M | C: 68478, D:00018224 N: N0000178379,  DB01590 | 26868298 |
| Fangchinoline | O | C: 132893 | 31690059 |
| Favipiravir | S2 | C: 134722, DB12466 | 32020029;  Doi:10.1101/2020.03.17.20037432 |
| Gemcitabine hydrochloride | S, M | C: 31647, N: N0000022977, DB00441 | 24841273 |
| Geranylgeranylacetone (GGA) | S | C: 31649 | 30711575 |
| Gilteritinib | S | C: 145372, D: 00837869, DB12141 | 32366720 |
| Glycyrrhizin (  Glycyrrhizic acid) | S | C: 29807, D: 00723537, DB13751 | 21762538, 16837072 |
| Hydroxychloroquine sulfate | S, M | D: 00061833, N: N0000146583, DB01611 | 32150618, 24841273 |
| Indinavir | S | D: 00013621, DB00224 | 15144898 |
| Ivacaftor | S | C: 66901, D: 00020190, DB08820 | 32366720 |
| Ivermectin | S2 | C: 6078, N: N0000148510, D: 00020654, DB00602 | 32251768, 21297106 |
| Lopinavir | S, M | C: 31781, D: 00016421, N: N0000148672, DB01601 | 27344959, 26868298  16837072 |
| Lycorine | O, M, L, V | C: 6601 | 30918074 |
| Mefloquine | S, M | C: 63609, D: 00022383, N: N0000147900,  DB00358 | 19258267, 24841273 |
| Mycophenolate mofetil | O, M, L, V | C: 8764, D: 00013779, N: N0000148406,  DB00688 | 30918074 |
| Mycophenolic acid | M | C:168396, D:00016769, N: N0000148832, DB01024 | 27344959 |
| Niclosamide | S | C: 7553 D: 00015957, N: N0000146594,  DB06803 | 32366720, 32361588 |
| Nitazoxanide | M, S2 | C: 94807, D: 00015413, N: N0000148784,  DB00507 | 30918074, 25108173 |
| Nocodazole | M | C: 34892, N: N0000166936, DB08313 | 27783035, 24841273 |
| Omacetaxine mepesuccinate | S, M | C: 71019, D: 00018264, DB04865 | 24841273 |
| Oxyclozanide | S | N: N0000166893 | 32366720, 30626902 |
| Penciclovir | S2 | C: 7956, N: N0000148462, D: 00013349, DB00299 | 32020029 |
| Pyrvinium pamoate (Pyrvinium) | O, M, L, V | C: 8688, DB06816 | 30918074 |
| Rapamycin (sirolimus) | M | C: 9168, D: 00024510, DB00877 | 25487801, 26868298 |
| Remdesivir | S, M, S2 | C: 145994, D: 00882939,DB14761 | 30849247, 32275812, 32020029 |
| Ritonavir | S, M | C: 45409, D: 00023321, N: N0000148436,  DB00503 | 27344959, 15226499  16837072 |
| Ribavirin | S, M, O | C: 63580, D: 00025187, N: N0000147496,  DB00811 | 27344959, 26868298  15200845, 16837072 |
| Salinomycin | S | C: 80025, DB11544 | 32366720, 30282713 |
| SB203580 | S, M | C: 90705 | 24699705, 27344959 |
| SG85 | S, M | C: 147346 | 25039866, 27344959 |
| Selumetinib | M | C: 90227, D: 00877833, DB11689 | 26868298 |
| Silvestrol | M | C: 66484 | 30711575 |
| Rimantadine | S | C: 49886, N: N0000021902, D: 00017006, DB00478 | 15288617 |
| Tetrandrine | S, O | C: 49, DB14066 | 32366720, 31690059 |
| Trametinib | M | C: 75991, D: 00750784, DB08911 | 26868298 |
| ***Modulate immune response*** | | | |
| Azithromycin | S2 | C: 2955, N: N0000148074, D: 00024808, DB00207 | 32533455 |
| Cepharanthine | S, O | C: 3546 | 32366720, 31690059 |
| Ciclesonide | S | C: 31397, D: 00019082, N: N0000176150,  DB01410 | 32366720 |
| Fangchinoline | O | C: 132893 | 31690059 |
| IFNα2a | M | C: 5937, D: 00016915, N: N0000020127,  DB00034 | 26868298, 25278221 |
| IFNβ1b | M | C: 5938, D: 00027247, N: N0000021905, DB00068 | 27344959, 15200845 |
| Wellferon | S | D: 00013637, DB00011 | 15200845 |
| Glycyrrhizin | S | C: 29807, D: 00723537 | 21762538, 16837072 |
| Nitazoxanide | M, S2 | C: 94807, D: 00015413, N: N0000148784,  DB00507 | 30918074, 25108173 |
| Tetrandrine | S, O | C: 49, DB14066 | 32366720, 31690059 |
| Tocilizumab | S2 | N: N0000180629, D: 00019607, DB06273 | 32350134 |
| ***Unknown mechanism*** | | | |
| Fluspirilene | S, M | D: 00014099, N: N0000167366, DB04842 | 24841273 |
| Thiothixene | S, M | C: 9571, D: 00012220, N: N0000148032,  DB01623 | 24841273 |
| Fluphenazine hydrochloride | S, M | C: 5126, D: 00059506, N: N0000146092,  DB00623 | 24841273 |
| Promethazine hydrochloride | S, M | C: 8462, D: 00061963, N: N0000146203,  DB01069 | 24841273 |
| Astemizole | S, M | C: 2896, D: 00017043, N: N0000147536, DB00637 | 24841273 |
| Chlorphenoxamine hydrochloride | S, M | C: 135288, DB09007 | 24841273 |
| Thiethylperazine maleate | S, M | C: 32216, N: N0000147331, DB00372 | 24841273 |
| Clomipramine hydrochloride | S, M | C: 3755, D: 00058882, N: N0000147612,  DB01242 | 24841273 |
| Monensin | M | C: 27617, N: N0000167131, DB11430 | 24841273 |
| Aescin | S | C: 2500 | 15226499 |
| Reserpine | S | C: 28487, D: 00023295, N: N0000145891, DB00206 | 15226499 |
| Phenazopyridine | O, M, L,V | C: 71416, D: 00016762, N: N0000147969,  DB01438 | 30918074 |
| Cetylpyridinium chloride (Cetylpyridinium) | O, M, L, V | C: 32915, D:00050843, N: N0000147354, DB11073 | 30918074 |
| Oligomycin | O,M, L, V | C: 25675, N: N0000168432 | 30918074 |
| Harmine | O, M, L, V | C: 28121, N: N0000167259, DB07919 | 30918074 |
| Conessine | O, M, L, V | C: 27965 | 30918074 |
| Loperamide | S, O, M | C: 6532, D: 00000908, N: N0000147893,  DB00836 | 30918074, 27344959, 32366720 |
| Proscillaridin | S | C: 32065, D: 00012193, N: N0000168447,  DB13307 | 32366720 |
| Hydroxyprogesterone caproate | S | C: 5812, D: 00064769, N: N0000145993, DB06789 | 32366720 |
| Anidulafungin | S | C: 55346, D: 00018854, N: N0000171752, DB00362 | 32366720 |
| Bazedoxifene | S | C: 135947, D: 00750789, DB06401 | 32366720 |
| Eltrombopag | S | C: 85010, D: 00019163, N: N0000177933,  DB06210 | 32366720 |
| Baicalin | S | C: 2981, N: N0000179808 | 15288617 |
| Emetine dihydrochloride hydrate | S, M | C: 146000 | 24841273, 29557770 |

** Notation: For targeted virus, M: MERS-CoV, S: SARS-CoV, S2: SARS-CoV 2, O: HCoV-OC43, L: HCoV-NL63, V: MHV-A59. For ontologies, C: ChEBI, D: DRON, N: NDF-RT. Noted that Azithromycin was newly added due to its very recent online publication on May 17, 2020 ^49^.

**Supplemental Table 2:** Anti-coronavirus antibodies annotated from the literature and clinical trials

| **Antibody name** | **Antigen** | **Efficacy test** | **Type** | **PMID or clinical trial IDs** |
| --- | --- | --- | --- | --- |
| ***Targeting SARS-CoV*** | | | | |
| S3.1 | spike protein | *in vitro*/ *in vivo* mouse | monoclonal | 15247913, 17620608 |
| CR3014 | spike protein | *in vitro*/*in vivo* ferrets | monoclonal | 15220038, 15650189, 17620608 |
| S230.15 | S1 RBD* | *in vitro* | monoclonal | 17620608 |
| m396 | S1 RBD | *in vitro* | monoclonal | 17620608 |
| 80R | S1 | *in vitro* | monoclonal | 14983044, 17620608 |
| 201 | S1 RBD | *in vitro* | monoclonal | 15655773, 17620608 |
| scFv B1 | S2 | *in vitro* | monoclonal | 15939399, 17620608 |
| ***Targeting MERS-CoV*** | | | | |
| m332 | spike protein | *in vivo* (rabbits) | monoclonal | 27344959 |
| 311B-N1 | spike protein | *in vivo* (rhesus macaques) | monoclonal | 27344959 |
| REGN3051 | spike protein | *In vitro*/*in vivo* (mouse) | monoclonal | 26315600 |
| REGN3048 | spike protein | *In vitro*/*in vivo* (mouse) | monoclonal | 26315600 |
| 4C2 | S1 RBD* | *in vitro*/*in vivo* (mouse) | monoclonal | 28855003, 26391698 |
| Mersmab | S1 RBD | *In vitro* | monoclonal | 28855003 |
| m336 | S1 RBD | *In vitro*/*in vivo* (mouse, rabbit) | monoclonal | 28855003 |
| m337 | S1 RBD | *In vitro* | monoclonal | 28855003 |
| m338 | S1 RBD | *In vitro* | monoclonal | 28855003 |
| MERS-4 | S1 RBD | *In vitro* | monoclonal | 28855003 |
| MERS-27 | S1 RBD | *In vitro* | monoclonal | 28855003 |
| hMS-1 | S1 RBD | *In vitro*/*in vivo* (mouse) | monoclonal | 28855003 |
| LCA60 | S1 RBD | *In vitro*/*in vivo* (mouse) | monoclonal | 28855003 |
| 3B11-N | S1 RBD | *In vitro*/*in vivo* (rhesus monkeys) | monoclonal | 28855003 |
| 2E6 | S1 RBD | *in vitro* | polyclonal | 26391698 |
| 2F9 | DPP4 | *In vitro* | monoclonal | 28855003 |
| 1F7 | DPP4 | *In vitro* | monoclonal | 28855003 |
| YS110 | DPP4 | *In vitro* | monoclonal | 28855003, 26315600 |
| anti-CD26 | DPP4 | *in vitro* | polyclonal | 26315600 |
| ***Targeting SARS CoV- 2*** | | | | |
| 47D11 | Spike protein | *In vitro* | monoclonal | 32366817 |
| S309 | Spike protein | *In vitro* | monoclonal | 32422645 |
| CB6 | RBD | *In vitro/* *in vivo (rhesus*  *monkeys)* | monoclonal | 32454512 |
| CA1 | ACE2/S protein | *In vitro* | monoclonal | 32454512 |
| 4A8 | N terminal domain (NTD) of the S protein | *In vitro* | monoclonal | 32571838 |

*RBD: spike receptor-binding domain.
